# Exercise-induced muscle glucose uptake in mice with graded, muscle-specific GLUT-4 deletion

**DOI:** 10.1002/phy2.65

**Published:** 2013-08-22

**Authors:** Kirsten F Howlett, Sofianos Andrikopoulos, Joseph Proietto, Mark Hargreaves

**Affiliations:** 1 School of Exercise and Nutrition Sciences, Deakin UniversityGeelong, Victoria, Australia; 2 Department of Medicine, Heidelberg Repatriation Hospital, The University of MelbourneHeidelberg, Victoria, Australia; 3 Department of Physiology, The University of MelbourneParkville, Victoria, Australia

**Keywords:** exercise, GLUT4, muscle glucose uptake

## Abstract

To investigate the importance of the glucose transporter GLUT-4 for muscle glucose uptake during exercise, transgenic mice with skeletal muscle GLUT-4 expression approximately 30–60% of normal (CON) and approximately 5–10% of normal (KO) were generated using the Cre/Lox system and compared with wild-type (WT) mice during approximately 40 min of treadmill running (KO: 37.7 ± 1.3 min; WT: 40 min; CON: 40 min, *P* = 0.18). In WT and CON animals, exercise resulted in an overall increase in muscle glucose uptake. More specifically, glucose uptake was increased in red gastrocnemius of WT mice and in the soleus and red gastrocnemius of CON mice. In contrast, the exercise-induced increase in muscle glucose uptake in all muscles was completely abolished in KO mice. Muscle glucose uptake increased during exercise in both red and white quadriceps of WT mice, while the small increases in CON mice were not statistically significant. In KO mice, there was no change at all in quadriceps muscle glucose uptake. No differences in muscle glycogen use during exercise were observed between any of the groups. However, there was a significant increase in plasma glucose levels after exercise in KO mice. The results of this study demonstrated that a reduction in skeletal muscle GLUT-4 expression to approximately 10% of normal levels completely abolished the exercise-induced increase in muscle glucose uptake.

## Introduction

During exercise, skeletal muscle glucose uptake increases up to 40–50 times the basal level, depending upon the exercise intensity and duration (James et al. 1985; Katz et al. 1986). Skeletal muscle glucose uptake comprises three important steps: glucose delivery from blood to muscle, sarcolemmal glucose transport, and irreversible phosphorylation to glucose 6-phosphate by hexokinase (Rose and Richter 2005). Sarcolemmal glucose transport during muscle contractions is facilitated by the glucose transporter isoform (GLUT-4), which is translocated from intracellular sites to the plasma membrane and t-tubules in response to contractile/exercise stimuli (Douen et al. 1990; Kristiansen et al. 1996; Roy and Marette 1996). It has been demonstrated that skeletal muscle 2-deoxyglucose uptake during muscle contractions is directly related to muscle GLUT-4 protein content (Henriksen et al. 1990), and that overexpression of GLUT-4 increases muscle glucose transport activity during tetanic, electrical stimulation (Hansen et al. 1995). In contrast, recent studies suggest that GLUT-4-mediated muscle glucose transport does not limit exercise-stimulated muscle glucose uptake, only doing so when glucose phosphorylation capacity is enhanced by overexpression of hexokinase (Fueger et al. 2004). In genetically modified mice lacking GLUT-4, either globally or specifically within skeletal muscle, 2-deoxyglucose transport into skeletal muscle was markedly blunted in response to in vitro contractions (Zisman et al. 2000) or dynamic exercise (Ryder et al. 1999; Fueger et al. 2007). GLUT-4-deficient mice also demonstrated increased muscle fatigability during electrical stimulation (Gorselink et al. 2002), which could have been due to either reduced muscle glucose uptake during contractile activity and/or the lower prestimulation muscle glycogen levels in GLUT-4-deficient mice (Gorselink et al. 2002).

Using the Cre/LoxP system, we have previously generated muscle-specific GLUT-4-deficient mice which are characterized by normal basal and insulin-stimulated muscle glucose uptake (Kaczmarczyk et al. 2003; Fam et al. 2012). During the course of producing these mice, we observed that the control Lox/Lox mice had higher muscle GLUT-4 levels than the Lox/Lox/Cre (GLUT-4 KO) mice, but that muscle GLUT-4 levels were lower than those in wild-type control mice. This is believed to be due to the knockdown phenomenon of the Neomycin resistance cassette used in these gene ablation studies. Importantly, this provided us with an opportunity to examine the effect of graded GLUT-4 deletion in skeletal muscle on glucose metabolism during exercise. Thus, the aim of this study was to examine the effect of moderate and severe muscle-specific GLUT-4 deletion on muscle glucose uptake during treadmill exercise in mice. We hypothesized that exercise-induced muscle glucose uptake would be abolished in mice with GLUT-4 deletion.

## Methods

### Animals

Mice with specific deletion of GLUT-4 in skeletal muscle (KO) on a C57BL/6 × 129/Sv background generated using Cre-Lox methodology, control mice containing only Lox sites (CON) and wild-type C57BL/6 (WT) animals were studied at approximately 10 weeks of age. The generation of these animals and many of the physiological characteristics of the mice have been described in detail previously (Kaczmarczyk et al. 2003; Fam et al. 2012). Genotyping was performed by PCR using genomic DNA isolated from the tail tip of 3- to 4-week-old mice. Animals were housed in The University of Melbourne, Department of Medicine Animal Research Facility under a 12-h light/dark cycle and room temperature kept constant at 22°C. Animals had free access to standard laboratory chow consisting of 74% carbohydrate, 6% fat, and 20% protein, by weight (Barastoc Pty, Pakenham, Vic., Australia) and water. All experimental protocols were approved by the Animal Experimentation Ethics Committee of The University of Melbourne.

### Experimental protocol

All mice were accustomed to the rodent treadmill (Exer-4; Columbus Instruments, Columbus, OH) by walking or running for 10 min per day for two consecutive days before the experiment. On the experimental day, mice were fasted for 4 h prior to the experiment and then randomly divided into sedentary and exercise groups. Sedentary animals rested in their cages for 40 min. For the exercise group, mice ran on the treadmill at a speed of 20 m·min^−1^ and 10% incline for 40 min. This is a relatively high exercise intensity, estimated to be approximately 80% of maximal oxygen consumption (Fernando et al. 1993). Immediately after exercise or the resting period animals were killed by decapitation and trunk blood was collected. Blood was spun and the plasma removed and stored at −20°C for later analysis. Hindlimb (soleus, red and white gastrocnemius) and thigh (red and white quadriceps) muscles were quickly dissected and frozen in liquid nitrogen. The tissues were stored at −80°C for later analysis.

### Tissue glucose uptake

For measurement of individual tissue glucose uptake a modified labeled 2-[1-^14^C]-deoxyglucose technique was employed (Kraegen et al. 1985). All animals prior to rest or exercise were injected intraperitoneally with 50 μCi of 2-[1-^14^C]-deoxyglucose. We did not measure 2-^14^C-deoxyglucose in blood and assumed that systemic delivery of the tracer was similar in all animals. Muscles collected immediately following rest or exercise were then analyzed for muscle accumulation of phosphorylated 2-^14^C-deoxyglucose. In brief, a portion of muscle was weighed and digested in 1 mol·L^−1^ NaOH at 60°C for 1 h. Following digestion, the reaction was neutralized by 1 mol·L^−1^ HCl. The digest was then separated into two aliquots. To measure unphosphorylated 2-^14^C-deoxyglucose, an aliquot was deproteinized in equal volumes of Ba(OH)_2_ and ZnSO_4_ and spun. The supernatant was recovered and counted (LS CA 3801; Beckman Instruments, Irvine, CA). The second aliquot was deproteinized in 6% perchloric acid and spun. The supernatant was recovered and counted to determine total (phosphorylated and unphosphorylated) 2-^14^C-deoxyglucose. Muscle accumulation of phosphorylated 2-^14^C-deoxyglucose was then calculated as the difference between the aliquots, corrected for muscle weight.

### Plasma glucose, lactate, and insulin

Plasma glucose and lactate concentrations were measured using an automated analyzer (EML105; Radiometer, Copenhagen, Denmark). Plasma insulin concentrations were measured by radioimmunoassay (Linco Research, St Charles, MO).

### Tissue glycogen

For measurement of muscle (except quadriceps due to insufficient material) and liver glycogen content, a small portion of tissue was freeze dried, dissected free of visible connective tissue and blood, and then powdered. Samples were analyzed for glycogen content according to the method of Passonneau and Lauderdale (Passonneau and Lauderdale 1974).

### GLUT-4 protein

GLUT-4 protein content was measured in selected muscles of GLUT-4-deficient, control, and wild-type mice using Western blotting and a rabbit polyclonal antibody specific for GLUT-4, with appropriate loading controls, as described previously (Fam et al. 2012). There was insufficient material to measure GLUT-4 in soleus muscles.

### Statistical analysis

Data are expressed as mean ± SEM. Statistical analysis was undertaken using one- or two-way ANOVA. When ANOVA revealed significant differences, further analysis was performed using Tukey's post hoc test for multiple comparisons. Differences between groups were considered significant when *P* < 0.05.

## Results

### Animal characteristics

GLUT-4-deficient, control, and wild-type mice of both genders were studied at approximately 10 weeks of age. The body masses of the GLUT-4-deficient and control mice were lower (*P* < 0.05) compared with the wild-type mice (KO, 23.5 ± 1.3 g; CON, 24.5 ± 1.3 g; WT, 28.9 ± 0.9 g). As there were no gender differences in any of the parameters measured, data from male and female mice were pooled. All wild-type and control animals were able to complete the 40 min of treadmill exercise. Three of the 11 GLUT-4-deficient mice studied were unable to complete the required exercise bout, fatiguing after 27, 33, and 35 min; however, on average, there were no differences in exercise times among the three groups (KO: 37.7 ± 1.3 min; WT: 40 min; CON: 40 min, *P* = 0.18).

### Tissue GLUT-4 expression

On average, GLUT-4 was reduced by 85–95% in the gastrocnemius and quadriceps muscles of GLUT-4-deficient mice (Table 1). In control mice, GLUT-4 protein content was reduced 35–70% in gastrocnemius and quadriceps muscles (Table 1).

**Table 1:** GLUT-4 protein expression in selected tissues from wild-type (WT), control (CON), and GLUT-4-deficient (KO) mice

Tissue	WT	CON	KO
Red gastrocnemius	115 ± 4	46 ± 7*	16 ± 3*,**
White gastrocnemius	78 ± 5	24 ± 4*	7 ± 2*,**
Red quadriceps	76 ± 3	48 ± 4*	4 ± 0*,**
White quadriceps	65 ± 5	29 ± 4*	5 ± 2*,**

Data (arbitrary units) are means ± SEM (*n* = 6–11 mice per group).

* *P* < 0.05 compared with WT;

** *P* < 0.05 compared with CON.

### Metabolites, insulin, and glycogen

**Figure 1:**
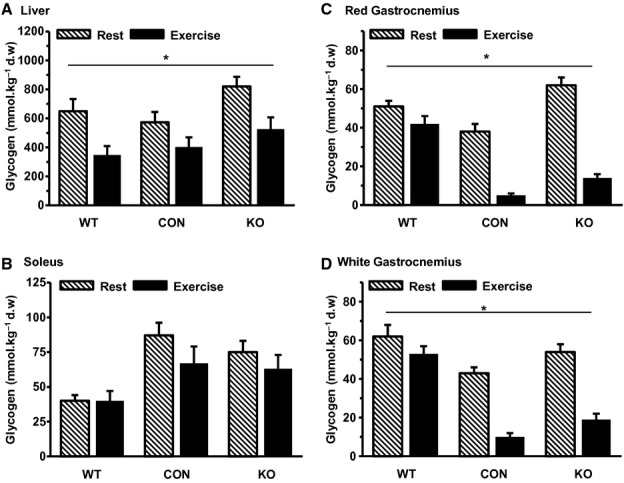
Liver and muscle glycogen at rest and following exercise in wild-type (WT), control (CON), and GLUT-4-deficient (KO) mice. Data are means ± SEM (*n* = 6–11 mice per group). d.w. denotes dry weight. *P < 0.05 (main effect) between Rest and Exercise.

Plasma glucose levels were similar in wild-type, control, and GLUT-4-deficient mice after a 4-h fast (Table 2). In response to exercise, plasma glucose levels were significantly elevated in the GLUT-4-deficient mice, only. Resting plasma insulin levels were variable among the three groups of mice, but in response to exercise plasma insulin levels decreased (*P* < 0.05, main effect) in all groups (Table 2). Plasma lactate levels were higher (*P* < 0.05, main effect) in the wild-type animals compared with the control and GLUT-4-deficient mice (Table 2). In all groups of animals, exercise did not significantly alter plasma lactate levels. At rest, liver and muscle glycogen levels were similar among all groups of animals (Fig. 1) and in response to exercise, glycogen levels decreased (*P* < 0.05, main effect) in all tissues, except the soleus (Fig. 1).

**Table 2:** Plasma glucose, lactate, and insulin concentrations at rest and following exercise in wild-type (WT), control (CON), and GLUT-4-deficient (KO) mice

	Rest	Exercise
Glucose (mmol·L^−1^)		
WT	11.7 ± 1.1	13.8 ± 1.5
CON	12.6 ± 1.9	9.6 ± 1.4
KO	11.2 ± 0.5	26.7 ± 3.5*,**
Lactate (mmol·L^−1^)		
WT	3.5 ± 0.4***	4.5 ± 0.3***
CON	2.7 ± 0.3	2.7 ± 0.2
KO	2.3 ± 0.2	2.2 ± 0.2
Insulin (pmol·L^−1^)		
WT	31.4 ± 5.7	22.9 ± 2.0****
CON	71.1 ± 3.8	53.0 ± 7.4****
KO	58.7 ± 15.3	37.1 ± 7.4****

Data are means ± SEM (*n* = 6–11 mice per group).

* *P* < 0.05 compared with Rest;

** *P* < 0.05 compared with WT and CON;

*** *P* < 0.05 (main effect) compared with CON and KO;

**** *P* < 0.05 (main effect) compared with Rest.

### Tissue glucose uptake

**Figure 2:**
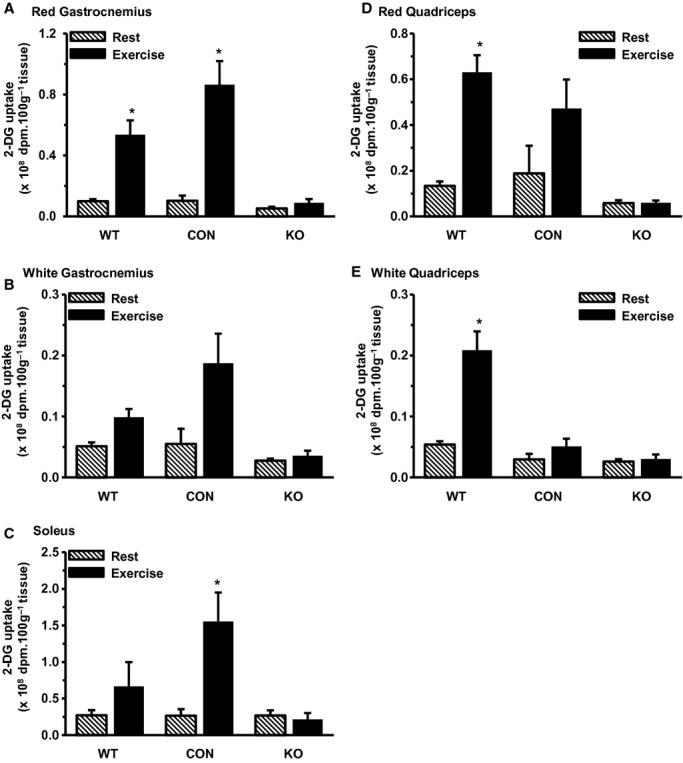
Muscle glucose uptake at rest and following exercise in wild type (WT), control (CON), and GLUT-4-deficient (KO) mice. 2-DG denotes 2-deoxyglucose uptake. dpm denotes disintegrations per minute. Data are means ± SEM (*n* = 6–11 mice per group). *P < 0.05 compared with Rest.

Under resting conditions, glucose uptake was similar in the hindlimb muscles from wild-type, control, and GLUT-4-deficient mice (Fig. 2). In wild-type and control animals, exercise resulted in an overall increase in muscle glucose uptake. More specifically, glucose uptake was increased (*P* < 0.05) in red gastrocnemius of wild-type mice and in the soleus and red gastrocnemius of control mice. In contrast, the exercise-induced increase in muscle glucose uptake observed in the wild-type and control mice was completely abolished in the GLUT-4-deficient mice (Fig. 2). Muscle glucose uptake increased during exercise in both red and white quadriceps of wild-type animals (Fig. 2), while the small increases in control mice were not statistically significant. In GLUT-4-deficient mice, there was no change at all in quadriceps muscle glucose uptake (Fig. 2).

## Discussion

The results of this study demonstrate that a approximately 90% reduction in skeletal muscle GLUT-4 completely abolished muscle glucose uptake during exercise; however, animals with a reduction in muscle GLUT-4 protein expression of 60–70% maintained their ability to increase glucose uptake during exercise. Thus, there appears to be a minimum level of GLUT-4 expression that is required for exercise-induced muscle glucose uptake. Despite the reduction in muscle glucose uptake during exercise in GLUT-4-deficient mice, they were, on average, able to complete the 40-min treadmill exercise task.

In this study, GLUT-4-deficient mice were generated using a Cre/LoxP system as described previously (Kaczmarczyk et al. 2003; Fam et al. 2012). One effect of this system was to cause “knockdown” of GLUT-4 in various muscles of Lox/Lox or control mice (Table 1). However, this enabled us to examine the effects of graded reductions in muscle GLUT-4 expression on exercise-induced muscle glucose uptake. Consistent with the findings of Fueger et al. (2007), a 60–70% reduction in muscle GLUT-4 expression did not impair the exercise-induced increase in muscle glucose uptake. This implies that there may be a significant “reserve” capacity of GLUT-4 within skeletal muscle and that only a relatively small fraction of total GLUT-4 is required for translocation during exercise in order for sarcolemmal glucose transport to be increased. In contrast, Zisman et al. (2000) observed graded reductions in muscle glucose transport in proportion to GLUT-4 expression in heterozygous (40–50%) and homozygous (95%) muscle-specific GLUT-4 knockout mice during in vitro muscle contraction. This may reflect a greater dependence on GLUT-4 for muscle glucose uptake in the in vitro model, differences in potential adaptive responses in the expression of other glucose transporters, and/or differences in experimental animals and models.

Of interest was the apparent increase in soleus and gastrocnemius glucose uptake during exercise in control compared with wild-type mice (Fig. 2). An explanation of this is not readily available; however, it is possible that muscle recruitment during exercise may have been different in the wild-type mice, thereby reducing reliance on hindlimb muscles. The trend for lower muscle glycogen use during exercise in wild-type animals (Fig. 1) provides some support for such a suggestion. Furthermore, muscle glucose uptake during exercise in wild-type animals was increased to a greater extent in red and white quadriceps muscles compared with hindlimb muscles (Fig. 2). Nevertheless, these observations do not change the major conclusion of this study, based on the difference between control and GLUT-4-deficient mice, that a minimum level of GLUT-4 is required for exercise-induced muscle glucose uptake. Exercise in GLUT-4-deficient mice produced a marked hyperglycemia, with plasma glucose being approximately 3 fold higher at the end of exercise (Table 2). While this has the potential to influence competition between endogenous glucose and the 2-deoxyglucose tracer, and our subsequent tissue glucose uptake measurements, the maximum effect would be to increase tracer-determined tissue glucose uptake in GLUT-4-deficient mice by a factor of three, assuming that the muscle was able to more effectively take up endogenous glucose under such conditions. However, this may not be the case as the lack of GLUT-4 should have decreased the ability of muscle to take up both endogenous glucose and the 2-deoxyglucose tracer. We believe that the hyperglycemia is a consequence, not a cause, of the lower tracer-determined muscle glucose uptake in GLUT-4-deficient mice, reflecting an impaired ability for glucose uptake in skeletal muscle during exercise.

All but three of the 11 GLUT-4-deficient mice studied were able to complete the 40-min treadmill exercise task. Somewhat surprisingly, there was no greater muscle glycogen use during exercise in GLUT-4-deficient mice (Fig. 1). However, it has recently been demonstrated that muscle glycogen is not essential for strenuous exercise performance in mice (Pederson et al. 2005). Thus, although we have no direct measurements of lipid metabolism, it would appear that the GLUT-4-deficient mice were able to compensate for reduced muscle glucose uptake by increasing reliance on lipid fuel sources. Muscle glycogen levels have been reported to be higher in mice with skeletal muscle-specific GLUT-4 deletion (Kim et al. 2005), although we observed no differences in this study. There are reports of reduced treadmill exercise performance (Fueger et al. 2007) and increased muscle fatigability (Gorselink et al. 2002) in mice lacking GLUT-4 in skeletal muscles. Although, on average, exercise time was not different among the three groups of mice in this study, three of the KO mice were unable to complete the entire 40-min treadmill exercise, suggesting that these mice may be more susceptible to accelerated fatigue during exercise.

In summary, the results of this study demonstrated that a modest (approximately 30–60%) reduction in skeletal muscle GLUT-4 expression did not affect skeletal muscle glucose uptake during exercise, while a reduction to approximately 10% of normal levels completely abolished the exercise-induced increase in muscle glucose uptake.
